# Use of the LENA Autism Screen with Children who are Deaf or Hard of Hearing

**DOI:** 10.3390/medicina55080495

**Published:** 2019-08-16

**Authors:** Mark VanDam, Christine Yoshinaga-Itano

**Affiliations:** 1 Department of Speech & Hearing Sciences, Elson S. Floyd College of Medicine, Washington State University, Spokane, WA 99202, USA; 2 Hearing Oral Program of Excellence (HOPE), Spokane, WA 99202, USA; 3 Institute of Cognitive Science, University of Colorado, Boulder, CO 80309, USA

**Keywords:** deaf, hard of hearing, D/HH, childhood hearing loss, autism spectrum disorder (ASD), automatic language screen, child language, LENA

## Abstract

*Background and Objectives*: This systematic review reports the evidence from the literature concerning the potential for using an automated vocal analysis, the Language ENvironment Analysis (LENA, LENA Research Foundation, Boulder, CO, USA) in the screening process for children at risk for autism spectrum disorder (ASD) and deaf or hard of hearing (D/HH). ASD and D/HH have increased comorbidity, but current behavioral diagnostic and screening tools have limitations. The LENA Language Autism Screen (LLAS) may offer an additional tool to disambiguate ASD from D/HH in young children. *Materials and Methods:* We examine empirical reports that use automatic vocal analysis methods to differentiate disordered from typically developing children. *Results:* Consensus across the sampled scientific literature shows support for use of automatic methods for screening and disambiguation of children with ASD and D/HH. There is some evidence of vocal differentiation between ASD, D/HH, and typically-developing children warranting use of the LLAS, but additional empirical evidence is needed to better understand the strengths and weaknesses of the tool. *Conclusions:* The findings reported here warrant further, more substantive, methodologically-sound research that is fully powered to show a reliable difference. Findings may be useful for both clinicians and researchers in better identification and understanding of communication disorders.

## 1. Introduction

The traditional definition of autism spectrum disorder (ASD) [1] does not include children who are deaf or hard of hearing (D/HH), nor children with significant intellectual disability, although specifiers and modifiers are used to indicate comorbidities and known etiologies, including hearing loss (HL). This population of D/HH children may be at higher risk for an additional disability of ASD due to increased time in the newborn intensive care unit, a known risk factor for autism [2]. However, this population of children with hearing loss is in dire need of a screening tool for ASD that will aid in earlier differential diagnosis of ASD and earlier treatment, particularly in the first three years of life.

### 1.1. Prevalence of Autism Spectrum Disorder in Children with Normal Hearing

All prevalence data reported here are from sources citing data in the United States. The most recent (2014) comprehensive report on the prevalence of ASD in children reported by the Centers for Disease Control is 1 in 59 children at 8 years of age [3]. The reported prevalence has been increasing, but it is not known whether or not there is better reporting and diagnosis or whether the actual prevalence has increased [4,5]. Large differences by sex have been reported. One in 42 boys as compared to one in 189 girls were identified with ASD. The median age of ASD identification was 52 months and did not differ by sex or race/ethnicity. Non-Hispanic black children were 30% less likely to be identified with ASD than non-Hispanic white children. Hispanic children were 50% less likely to be to be identified with ASD than non-Hispanic white children.

### 1.2. Prevalence of ASD in Children with Hearing Loss

The prevalence of autism in children with hearing loss is reported to be higher than for children with normal hearing [6,7]. There are not sufficient studies to determine the exact prevalence, but reports range from 4 to 9% of the population of children who are deaf or hard of hearing [8,9]. Even at the more conservative 4%, the prevalence would be about three times greater than for the general population. There is research that reports a higher incidence of ASD among children with bilateral profound hearing loss than any other category of hearing loss [6].

Jure and colleagues [7] found that about 4 in every 100 children with hearing loss had an additional diagnosis of ASD. Children with additional disabilities, such as intellectual and vision disabilities also have a higher prevalence of ASD. One-fifth of the children in this study had intellectual ability within the normal range and one-fifth had severe intellectual disability. The sample studied were children whose hearing loss was caused by rubella and meningitis. Today, these causes of deafness are infrequent, while cytomegalovirus (CMV) remains a significant cause of congenital as well as acquired/progressive hearing loss [6]. There is also a higher reported incidence of ASD among deaf/hard of hearing and children with visual impairment [9], and one study looked at the association between ASD and visual impairment compared with ASD and D/HH [10] finding that 19% of visually impaired children also had ASD and 9% of D/HH children had ASD.

### 1.3. Co-Morbidities between ASD and Hearing Loss

A number of conditions have been linked to both hearing loss and ASD (or characteristics consistent with an ASD) including rubella, cytomegalovirus (CMV), herpes, prematurity, toxoplasmosis, CHARGE syndrome, meningitis and measles [7,11,12].

### 1.4. Late Identification of Hearing Loss in Children

Children with late-identified hearing loss have behaviors that have been described as “autistic-like” because when children who are deaf or hard of hearing fail to have access to language, they may exhibit a high proportion of non-speech vocalizations (e.g., shrieks, cries, screams), may fail to turn-take vocally in conversations, may exhibit colliding speech with their conversational partners, may show self-destructive behaviors of frustrations (e.g., biting, head banging, or other self-harming behaviors), and may express increased frustration with peers and adults (e.g., hitting, biting, punching, throwing their bodies). As a result, screening and diagnostic assessment tools for the identification of ASD, such as the gold-standard Autism Diagnostic Observation Schedule (ADOS) [13], caution that the test should not be used with children who are deaf or hard of hearing or contain items that ask parents if their children act like they are deaf or have hearing loss. The ADOS in particular is not valid on children with significant sensory impairment, including children with hearing impairment [6].

### 1.5. Late Identification of ASD in Children with Hearing Loss

Compounding the significant impact of ASD is that most D/HH children are identified with ASD later than children with normal hearing [6,8,14,15].

### 1.6. Prevalence and Dynamic Population Characteristics

The foregoing descriptions of the populations of children with ASD and HL have changed substantially in recent years due to changing testing procedures and techniques, intervention strategies—importantly including newborn hearing screening and technologies such as cochlear implants and digital hearing aids, and trends in education, public policy, and healthcare [16,17]. In general, the breadth and application of intervention has increased, and outcomes have been demonstrably effective [18]. As mentioned above, however, incidence in some domains has decreased (e.g., HL due to measles—the current vaccine opt-out related measles outbreaks notwithstanding [19]) while incidence in other domains has increased (e.g., diagnosis of ASD, with some debate between incidence and diagnosis rates). The current rate of measles in the US is an interesting case where incidence rates have risen sharply in some areas such as Clark County, Washington, due to unfounded fear that vaccinations for measles (and other preventable diseases) may have a causal relationship with the onset of ASD. Many studies have “definitively refuted any connection between vaccines and autism” [20], yet reluctance to vaccinate children—below 80% vaccination rates in Clark County, WA, for example [21]—has demonstrably higher risk of incidence of measles and, consequently, hearing loss. That is to say, reluctance to vaccinate due to fear of ASD will have no effect on ASD risk but has demonstrable increased risk of hearing loss (and other deleterious health effects including death). Because of these rapid, recent changes, the modern populations of children with HL and ASD are poorly understood and have only a weak relationship with now-dated evidence in the literature that describes a different population.

### 1.7. Consequences of ASD Co-Morbid with Hearing Loss

Undiagnosed or late-diagnosed ASD in children who are deaf or hard of hearing can have dire consequences resulting in severe emotional-behavioral violent and destructive behaviors, as well as low or non-existent communication skills. Lack of access to language increases undesired or aberrant behaviors [22,23] and can leave teachers and other professionals under-prepared [24]. These behaviors take precedence over other academic or communication needs when considering educational placement and services [25]. The likelihood that students will be able to participate in general education environments is markedly decreased [26,27,28] as well as the inability to integrate into the community [29].

### 1.8. Traditional Behavioral and Objective Diagnosis

Traditional diagnosis of ASD is done by extensive behavioral observation and professional testing. The most widely used test is the ADOS [13], which requires professional administration and interpretation. The ADOS can identify children with ASD as young as 18 months and has well-understood testing properties, although diagnosis is more stable at older ages and average diagnosis of ASD is around 4 years of age [30]. One issue discussed directly in the ADOS manual [13] and in much of the ASD literature is the difficulty with interpretation in the face of multiple sensory disorders such as hearing loss. The ADOS (and many other diagnostic measures) are nevertheless used because there is no alternative. There have been a number of scientific, evidence-based evaluations of the ADOS for children with hearing loss [31,32,33], but none have attempted to validate on a population sample that suitably generalizes. In general, regardless of comorbidities, it is uncontroversial that earlier diagnosis of ASD (and other sensory disorders including hearing loss) is an advantage for the child (and family) and promotes development and positive outcomes. Early diagnosis of ASD, within the first three years of life, has resulted in a reduction in the severity of the diagnosis, the development of communication skills, and, for some children, ability to be educated in the typical, mainstream classroom.

Another tool used by practitioners and researchers is the Baby and Infant Screen for Children with aUtIsm Traits (BISCUIT). One study looked at toddlers with diagnosed ASD (and other similar sensory disorders) and examined whether the BISCUIT could aid in differentiating ASD from D/HH characteristics [34]. This study examined children with conductive HL, while the majority of studies in the literature report samples with permanent HL. Results from this study suggest differentiation is possible, but only after definite diagnosis has been achieved with other methods (in this study, via the BDI-2, M-CHAT, and professional experience). This study simultaneously demonstrates the ability to differentiate ASD from D/HH in toddlers on the one hand, and a need to have independent screening tools on the other hand.

In addition to behavioral testing described above, automated screening (and potentially future diagnostic) tools would serve the community at risk for ASD both alone and as comorbid with hearing loss. One tool is the LENA system. The system uses a passive, body-worn audio recorder that collects audio from the perspective of a young child in units of a day, up to 16 h of audio. The collected raw audio is uploaded to a computer and processed with the LENA software to determine the presence and frequency of speech, communicative, and acoustic features known to be developmentally important. The software then outputs summary statistics, a diarized report, the entire daylong audio, and other details. One of those tools is the LENA Language Autism Screen (LLAS) [35] that assesses natural daylong audio recordings collected in situ in the participants’ homes. Automatic speech processing (ASP) assesses speech, language, and communicative features known to be important for language development and known to be associated with higher risk of a disorder [36]. The software then compares the features from the sampled audio to a population sample of typically-developing children. The principle advantages of automated processing include cost, non-invasiveness, potential for remote or tele-administration, and objectivity of results [37].

According to several recent studies, this specific population of dual or multiple diagnoses is in dire need of a screening tool for ASD that will aid in earlier diagnosis of ASD and earlier treatment, particularly in the first three years of life [14,31]. Without the ability to screen, refer and diagnose, it is not possible to design or assess the efficacy of interventions for this population of children. Therefore, it is critical to identify reliable and effective screening tools for ASD within the first years of life.

### 1.9. Objectives

This systematic review will investigate the potential of automatic vocal analysis as an effective screening tool for ASD in young children that can be used in combination with other behavioral screening tools for infants and toddlers who are deaf or hard of hearing. The following questions guide this systematic review.

1. What is the evidence that the Language ENvironment Analysis (LENA) Language Autism Screen (LLAS) has demonstrated sensitivity, specificity, positive predictive value, negative predictive value, and overall accuracy to promote the use of the LLAS for the general population?
2. What is the evidence that key variables in the LLAS can differentiate typically developing children from children with ASD, language delays, and hearing loss?
3. What is the evidence on the use of the LLAS as a screen for autism with children who are deaf or hard of hearing?

## 2. Materials and Methods

The Preferred Reporting Items for Systematic Reviews and Meta-Analyses (PRISMA) protocol was used to design the present meta-analysis [38].

### 2.1. Protocol and Registration

The present work was registered with PROSPERO under registration receipt number 138195 available on the web at https://www.crd.york.ac.uk/prospero/.

### 2.2. Eligibility Criteria

We used the following inclusion criteria to determine the scope and range of scientific reports to include in this review.

Dissemination characteristics:

1. The publication period must be 2004–2019 because the LENA technology was not available prior to 2004.
2. The report must be archived, empirically-based, peer-reviewed, published (a) journal articles, (b) books or book chapters, (c) abstracts from conference presentations, (d) presentations, or (e) technical manuals. The inclusive scope of is intentionally broad to include work that was disseminated at professional conferences in order to capture detail of a rapidly changing, multidisciplinary technology. Reports accepted for publication (e.g., “accepted,” “in press,” and the like) are included, but reports not yet accepted in their final form (e.g., “in preparation,” “submitted,” and the like) are not included.
3. Publications must be in English.
4. In the case of duplicate reports, only the most recent was used. For example, if a report of equivalent scientific content were presented at two professional meetings, only the most recent report is included.

Content characteristics:

1. The participants must speak English or primarily English because the LENA automatic software processing was developed using English.
2. The study must report use of automated vocal analysis technology for screening.
3. The study must report a target group of children with ASD and one or more comparison target groups, identified as typically developing, language delayed, deaf/hard of hearing, or other co-morbid conditions.

### 2.3. Information Sources and Search Strategy

OneSearch, Medline, ERIC, Psych Abstracts, dissertation/thesis archives, LENA Foundation archives, and references from relevant published articles were examined. Some relevant work from published “works cited” led to archival records of conference presentations via researcher or author websites, and those sources were considered to be available.

The key search terms “autism”, “LENA”, “autism + screen”, “infant”, “toddler”, “deaf”, and “hard + of + hearing” were used. It should be noted that the search term “LENA,” occasionally in combination with another target word, yielded results primarily associated with the personal name of an author. These results were screened and are not included in the present review.

### 2.4. Study Selection, Data Collection Process, and Data Items

Two reviewers independently examined the reports from the search results and selected the articles that meet the inclusion characteristics. Data extraction from articles and presentations includes information about the study participants, the statistical methods, and the results of each study. If available, tables, charts, and figures were examined. Data items examined and collected from the reports are the sensitivity, specificity, positive predictive value, negative predictive value, and Cohen’s Kappa.

### 2.5. Risk of Bias in Individual Studies and Across Studies

Given the relative recency of this technology, the present work is a comprehensive study that attempts to capture all known studies meeting inclusion criteria. Additional contributions to the literature may examine known effects of socio-economic status (SES), influence of native language, age at diagnosis or identification (of hearing loss, ASD, or other comorbid conditions), family and demographic characteristics, genotypic influences, interactions with therapy or interventions, or sex of the child. Although some recent reports in the literature have examined the use of automatic speech technology in the developing or pre-industrial world [39], the overwhelming majority of the extant literature are reports of Western families with, ceteris paribus, the population advantages of modern, Western interventions, technology, and resources.

One known limitation of all studies is that the age range was birth through 48 months because LENA norms do not extend beyond 48 months. Due to widespread use of newborn hearing screening and Early Hearing Detection and Intervention (EHDI) programs [16], hearing loss is typically identified in the first few months of life [40,41], but ASD and other conditions/disorders are typically, and necessarily in some cases, identified later in development [42]. These disorders thus may benefit from an extended screening frame. Another limitation is that the participant samples are not random. For example, ASD samples in some cases were matched with a typically-developing (TD) sample in order that variables such as chronological age, sex, and race/ethnicity did not interfere or co-vary with comparability. In other cases, samples of convenience or opportunity allowed for comparison in a post-hoc sense, but precluded a rigorous, randomized design of specific participants. Finally, as mentioned above, both ASD and D/HH populations have distributional characteristics in the population that do not randomly present (for example, higher co-morbidity, genetic influence, or external interactions such as SES).

### 2.6. Summary Measures and Synthesis of Results

The studies included in this systematic review are given in Table 1 below with study details.

## 3. Results

**Figure 1:**
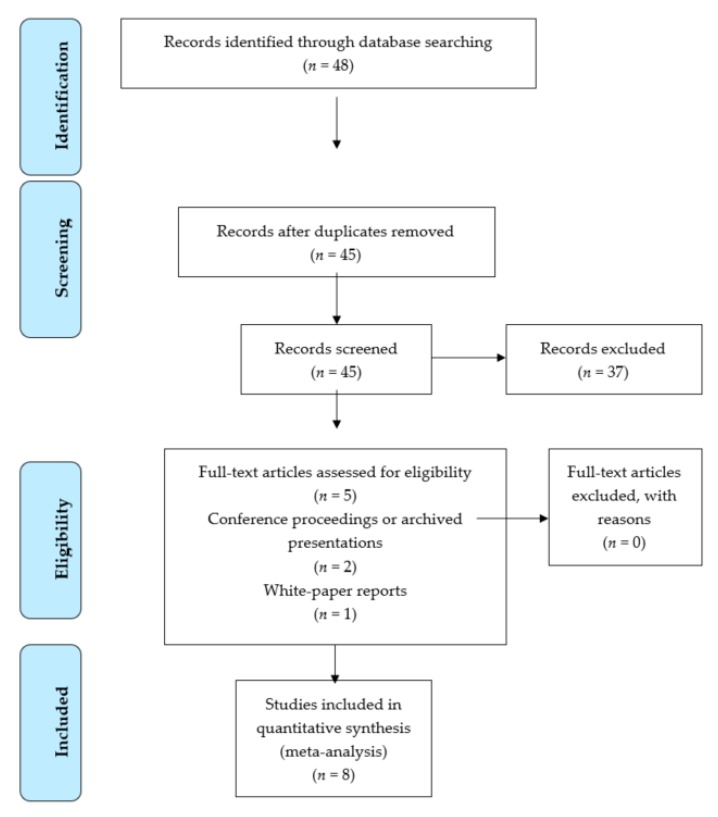
Flow chart of the present study.

A total of 48 studies were retrieved from the databases (Figure 1). After excluding duplicate studies, 45 were screened and 37 or those were excluded. Studies were excluded for not meeting the following eligibility criteria in §2.2 above: Participants with hearing loss but not ASD were described (*n* = 7), participants with disorders other than ASD or hearing loss were described (*n* = 9), participants with ASD were described but no controls were included (*n* = 9), study was not reported in English (*n* = 2), automatic vocal analysis methods were not used (*n* = 8), the study was not empirical or qualitative (2). In total, 8 studies are included in this review. Of those 8, five are traditional journal articles, two are archived conference presentations, and one is a white paper archived on the LENA Foundation website.

Results are given in Table 2 below. Overall, all studies achieved the ability to discriminate groups based on automatic vocal analysis. Studies employed different analytic methods (described in Table 2 below) that highlight aspects of each study to answer the individual research questions. Two of the included studies report sensitivity and specificity rates. The sensitivity—the ability to identify true positive group identification—was reported as 75 and 86. The specificity—the ability to identify true negative group identification—was reported as 98 and 86. These results appear to be relatively good indicators that the automatic software is doing a reasonable job to classify children with ASD, D/HH, and TD using objective, passive methodology.

## 4. Discussion

### 4.1. Summary of Evidence

Overall, there is good evidence that automatic vocal analysis methods are able to play a role in screening for communicative disorders, and those methods may identify children with ASD from children with hearing loss. There is also some evidence that automatic methods may be useful in screening or assessment of other disorders, including potentially co-morbid communicative disorders such as language delay. Here we describe the potential for a modern technology, the LLAS, to supplement other forms of identification, diagnosis, and screening for children at risk for communicative disorders. Although the technology is currently developing, early results are promising and the evidence in the literature suggests potential for applications to this sensitive population.

### 4.2. Limitations

The present work is limited by a relatively small sample size of studies looking at using automatic methods to discriminate among TD, ASD, and other groups. Here we restricted our search space to academic databases known to contain relevant empirical work. Additional empirical work may be collected from other sources, including conference presentations and workshops. As the technology becomes more widespread, and as it further develops and improves, additional empirical evidence in the literature will achieve a fuller picture of the use of automatic methods. Other areas for improvement include application of the technology in languages other than English and in non-Western and other cultural contexts. There are, for example, well-known interactions between social factors and language development [49], including specifically with automatic methods such as the LENA [50]. There are also some emerging analyses of using automatic methods with children who speak Russian, Arabic, and Mandarin. Results from those studies may shed light not only on linguistic and cultural differences but also on shared characteristics of children at risk for or diagnosed with speech and language disorders [51,52,53,54]. Finally, an alternative to the LENA processing strategies may offer additional diagnostic options, transparency, and reduce the cost of implementation [55]. It is also clear that this technology is not a diagnostic tool, but is best used as a screening tool in conjunction with other validated clinical tolls and professional expertise.

This work is descriptive of the extant literature on the topic. As more empirical work becomes available and more data becomes available through initiatives such as HomeBank (https://homebank.talkbank.org/) [56], opportunities for meta-analyses and larger-scale studies will become available. This will allow for more generalized cross-group comparisons with the potential to sort out differences among and within groups and sub-groups.

## 5. Conclusions

Here we reviewed the literature reporting automatic methods of vocal analysis used for disambiguation among participants with ASD and D/HH. Because ASD and D/HH may present similarly, it is crucial to identify via differential diagnosis. The automatic tools reviewed here may provide an especially useful, non-invasive screening tool for the clinician to assist in providing diagnosis or services. The findings to date in the literature are promising, but more research is needed to warrant widespread application in a variety of settings such as with comorbidities, social settings, languages other than English, and at ages not yet explored in the literature.

The technology of automatic vocal analysis has been accelerating recently and its application has become more widespread in academic, commercial, and therapeutic contexts [55]. The methodological approach common to the work here is the future of communication disorders, including its application and potential in tele practice [57], developing communities, and in the modern provision of services to a diverse, dynamic population.

## Figures and Tables

**Table 1:** Characteristics of the studies included in the systematic review (*n* = 9). Abbreviations: LENA: Language ENvironment Analysis; ASD: autism spectrum disorder; TD: typically developing; D/HH; deaf or hard of hearing; HH: hard of hearing; LD: language delayed; AWC; Adult Word Count; M-CHAT: Modified Checklist for Autism in Toddlers; SCQ: Social Communication Questionnaire; CBCL: Child Behavior Checklist; CSBS: Communication and Symbolic Behavior Scales; LDS: Language Development Survey; BRIEF-P: Behavior Rating Inventory of Executive Function—Preschool version; MBCDI, MacArthur Bates Communicative Development Inventories; PLS-4: Preschool Language Scale; CDI: Child Development Inventory; CAT/CLAMS: Cognitive Adaptive Test/Clinical Linguistic and Auditory Milestone Scale; GFTA: Goldman-Fristoe Test of Articulation; PPVT: Peabody Picture Vocabulary Test; REEL-3: Receptive-Expressive Emergent Language Test–Third Edition.

Study, First Author, Year	Stated Aim	Participants	Screening Tool/s	Participant Ages (Months)
Warren et al., 2010 [43]	Compare vocal productions and language learning environment.	28 ASD	LENA, MCHAT, SCQ, CBCL, CSBS, CDI, LDS, BRIEF-P, MB-CDI.	16–48
		78 TD		
VanDam et al., 2015 [44]	Do LENA measures predict group classification?	106 TD	AWC, Child Vocalizations	11–48 HH
		44 HH		10–48 TD
		49 LD		10–44 LD
		77 ASD		16–48 ASD
Oller et al., 2010 [36]	What is the sensitivity and specificity of automated autism screen for classification?	106 TD	Automated Autism Screen	10–48 TD
		77 ASD,		10–44 LD
		49 LD		16–48 ASD
Xu et al., 2009 [45]	Algorithm development for autism screen.	35 ASD	LENA, M-CHAT, SCQ, PLS-4	24–48
		30 LD		
		76 TD		
Richards et al., 2010 [35]	Demonstrate sensitivity, specificity, positive predictive value, negative predictive value	77 ASD	BRIEF-P, CDI, CBCL/LDS, CSBS-CQ M-CHAT, MBCDI, SCQ, LENA Snapshot, CAT/CLAMS, GFTA, PLS-4, PPVT, REEL-3	10–48
		106 TD		
		49 LD		
				8–48
Carr et al., 2012 [46]	Development of screening tool for ASD and DHH children.	3 ASD	LENA LLAS, CDI	0–72
		97 D/HH		
Xu et al., 2012 [47]	Can automatic methods classify children in known groups?	106 TD	Acoustic features: vowel (%, amplitude, duration, pitch, voicing), consonant (%), non-speech (spectral entropy)	8–48 TD
		71 ASD		16–48 ASD
		49 LD		10–48 LD
Xu et al., 2014 [48]	Validation of automatic methods and effectiveness in ASD screening.	106 TD	LENA LLAS	8–48 TD
		71 ASD		16–48 ASD
		49 LD		
				10–48 LD

**Table 2:** Characteristics of the studies included in the systematic review (*n* = 9). CT: conversational turns; AWC: adult word count; CM: child monologue; Dur: duration; ECCR: equal correct classification rate; PPT: posterior probability threshold; EER: equal error rate; PPV: positive predictive value; NPV: negative predictive value; HL: hearing loss; EEP: equal error probability.

Study, First Author, Year	Vocal Characteristics	Sensitivity, Specificity	Method(s); Discrimination or Difference	Ability to Discriminate Groups?
Warren et al., 2010 [43]	Conversational characteristics,	n.d.	*t*-test;	Yes
			CT: 26% more in TD	
			CV: 29% more in TD	
	CT, CV, AWC, CM, duration		AWC: n.s.	
			CM: 19% more in ASD	
			Dur: 24% more in TD	
VanDam et al., 2015 [44]	Developmental vocal age	n.d.	ECCR (%), PPT (%);	Yes
			HH: 76.4, 44.0	
			ASD: 85.7, 24.0	
			LD: 73.0, 38.0	
Oller et al., 2010 [36]	Articulatory features, developmental vocal age	Sens: 75	EEP (ratio);	Yes
			ASD v. TD: 86	
		Spec: 98	ASD+LD v. TD: 79	
Xu et al., 2009 [45]	Uni- and bi-phone phonetic analysis	n.d.	EER;	Yes
			77–83% (at recording)	
			85–90% (at child)	
Richards et al., 2010 [35]	CV, age	Sens: 86	EER, PPV, NPV, accuracy;	Yes
			88%	
			PPV: 0.79–0.91	
		Spec: 86		
			93–94%	
			88–92%	
Carr et al., 2012 [46]	LLAS	n.d.	Referral %	Yes
			HL-all: 18.1	
			HL-HF: 16.7	
			HL-UL: 11.1	
			HL-AN: 20.0	
			HL-MM: 14.5	
			HL-mod: 10.0	
			HL-sev: 22.2	
			HL-prof: 28.2	
Xu et al., 2012 [47]	Sound collision, vowel amplitude, child-directed voice	n.d.	DA, EER;	Yes
			Discriminability: 94%	
			EER: 6%	
			Sound collision: higher in ASD	
			Vowel amplitude: higher in ASD Child-directed: louder, longer duration, higher pitch, lower spectral entropy	
Xu et al., 2014 [48]	ACPU vowels and consonants	n.d.	ACPU-age correlation (consonant, vowel);	Yes
			TD: 63, 58	
			LD: 32, 19	
			ASD: 32, 25	

## Abbreviations

TD	typically developing;
ASD	autism spectrum disorder;
DHH	deaf/hard of hearing;
LD	language delayed;
LENA	Language Environment Analysis;
M-CHAT	Modified Checklist for Autism in Toddlers;
SCQ	Social Communication Questionnaire;
CBCL	Child Behavior Checklist;
CARS	Childhood Autism Rating Scale;
SRS	Social Responsiveness Scale;
CSBS	Communication and Symbolic Behavior Scales;
MSEL	Mullen Scales of Early Learning;
CDI	Child Development Inventory;
LDS	Language Development Survey;
BRIEF-P	Behavior Rating Inventory of Executive Function—Preschool version;
MBCDI	MacArthur Bates Communicative Development Inventories;
AWC	Adult Word Count;
BISCUIT	Baby and Infant Screen for Children with aUtIsm Traits;
BDIST	Battelle Developmental Inventory Screening Test;
PLS-4	Preschool Language Scale, Fourth Edition;
CAT/CLAMS	Cognitive Adaptive Test/Clinical Linguistic and Auditory Milestone Scale;
GFTA	Goldman-Fristoe Test of Articulation;
PPVT	Peabody Picture Vocabulary Test;
REEL-3	Receptive-Expressive Emergent Language Test–Third Edition;
LENA LLAS	Language ENvironment Analysis Language and Autism Screen;
CT	conversational turns;
AWC	adult word count;
CV	child vocalizations;
CM	child monologue;
Dur	duration;
ACPU	average count per utterance;
ECCR	equal correct classification rate;
PPT	posterior probability threshold;
n.d.	no data;
n.s.	not significant;
Sens	sensitivity;
Spec	specificity;
EEP	equal error probability;
EER	equal error rate;
PPV	positive predictive value;
NPV	negative predictive value;
DA	discriminant analysis;
ASD	autism spectrum disorder;
TD	typically developing;
LD	language delayed;
DHH	deaf/hard of hearing;
HL	hearing loss;
HL-HF	high-frequency hearing loss;
HL-UL	unilateral hearing loss;
HL-AN	auditory neuropathy syndrome disorder hearing loss;
HL-MM	multi-modal hearing loss;
HL-mod	moderate hearing loss;
HL-sev	moderate or severe hearing loss;
HL-prof	profound hearing loss
